# Borax and Iodide of Potash for the Voice

**Published:** 1884-11

**Authors:** 


### Borax and Iodide of Potash for the Voice.

A piece of borax the size of a pea dissolved in the mouth some ten minutes before speaking or singing strengthens the voice. Five grains of potassium iodide taken in a warm solution before going to bed the previous night, also helps the voice when an extra effort is required.—*Medical Herald.*

